# Cerebral Vasodilator Property of Poly(ADP-Ribose) Polymerase Inhibitor (PJ34) in the Neonatal and Adult Mouse Is Mediated by the Nitric Oxide Pathway

**DOI:** 10.3390/ijms21186569

**Published:** 2020-09-08

**Authors:** Philippe Bonnin, Christiane Charriaut-Marlangue, Julien Pansiot, Alexandre Boutigny, Jean-Marie Launay, Valérie C. Besson

**Affiliations:** 1 U1275, INSERM, F-75010, Physiologie Clinique—Explorations Fonctionnelles, Hopital Lariboisiere, Université de Paris, 2, Rue Ambroise Pare, 75010 Paris, France; alexandre.boutigny@aphp.fr; 2 U1141 NeuroDiderot, INSERM, Hôpital Robert Debré, Université de Paris, 48 Boulevard Sérurier, 75019 Paris, France; christiane.marlangue@gmail.com (C.C.-M.); julien.pansiot@inserm.fr (J.P.); 3 UMR-S942—Biomarqueurs Cardiovasculaires, Hopital Lariboisiere, Université de Paris, 2, Rue Ambroise Pare, 75010 Paris, France; jean-marie.launay@inserm.fr; 4 UMR-S1144—Optimisation Thérapeutique en Neuropsychopharmacologie, Faculté de Pharmacie de Paris, Université de Paris, 4 Avenue de l’Observatoire, 75006 Paris, France; valerie.besson@u-paris.fr

**Keywords:** cerebral blood flow, cerebral vasoreactivity, arterial dilation, mouse, brain, no synthase

## Abstract

The poly(ADP-ribose) polymerase (PARP) inhibitor PJ34 has been reported to improve endothelial dysfunction in the peripheral system. We addressed the role of PJ34 on the vascular tone and vasoreactivity during development in the mouse brain. Blood flows were measured in the basilar trunk using ultrasonography. Cerebral vasoreactivity or vasodilation reserve was estimated as a percentage increase in mean blood flow velocities (mBFV) recorded under normoxia-hypercapnia in control and after PJ34 administration. Non-selective and selective eNOS and nNOS inhibitors were used to evaluate the role of NO-pathway into the hemodynamic effects of PJ34. PJ34 increased mBFVs from 15.8 ± 1.6 to 19.1 ± 1.9 cm/s (*p* = 0.0043) in neonatal, from 14.6 ± 1.4 to 16.1 ± 0.9 cm/s (*p* = 0.0049) in adult, and from 15.7 ± 1.7 to 17.5 ± 2.0 cm/s (*p* = 0.0024) in aged mice 48 h after administration. These PJ34 values were similar to those measured in age-matched control mice under normoxia-hypercapnia. This recruitment was mediated through the activation of constitutive NO synthases in both the neonatal (38.2 ± 6.7 nmol/min/mg protein) and adult (31.5 ± 4.4 nmol/min/mg protein) brain, as compared to age-matched control brain (6.9 ± 0.4 and 6.3 ± 0.7 nmol/min/mg protein), respectively. In addition, quite selective eNOS inhibitor was able to inhibit the recruitment. PJ34 by itself is able to increase cerebral blood flow through the NO-pathway activation at least over 48 h after a single administration.

## 1. Introduction

Poly(ADP-ribose) polymerases (PARP) are a large family of constitutive nuclear enzymes that exert effects such as transcription regulation, cell division, and cell death [1,2].

In the central nervous system (CNS), several reports indicate the importance of PARP activation in numerous pathological situations. PARP inhibition [3,4], or PARP gene disruption [5], reduce infarct lesion preferentially in males with neonatal hypoxic-ischemia as well as with ischemia in young adult mice [6,7,8].

In the peripheral vascular system, the endothelial dysfunction consecutive to diabetes mellitus has been related to an endothelial depletion of NADPH, an essential cofactor of the endothelial nitric oxide (NO) synthase (eNOS) as demonstrated on isolated thoracic aorta [9]. Endothelial function is rapidly recovered by pharmacological inhibition of PARP. In addition, the endothelial dysfunction either associated with hypertension and aging [10] or induced by endothelin-1 (ET-1) [11] can be prevented by pharmacological inhibition of PARP with PJ34. Furthermore, the diminished vasoreactivity in ET-1-incubated vessels is improved by PJ34 [11].

The neurovascular unit (NVU) allows the relationship between brain parenchyma and its vessels. Under physiological conditions, and with an adequate supply of oxygen and nutrients, the vascular tone (balance between vasodilation and vasoconstriction) plays a role in the neurovascular coupling, leading to a direct link between neural activity and cerebral blood flow (CBF) (for review, see [12]). This vascular tone may be different in the developing brain as compared to adult brain, because vasoreactivity increases with developmental stages and the brain vasculature undergoes extensive endothelial proliferation and branching during the first postnatal month [13]. Moreover, mechanisms involved in vasoreactivity could differ and evolve with age. In the CNS, vascular dysfunction represents a major clinical problem after acute and chronic disease including stroke and neurodegenerative diseases. Based on the beneficial effects of PARP inhibitors to restore or enhance endothelial function in the peripheral vasculature and on the link between brain metabolism and arteriolar tone, we evaluated whether PJ34 could offer benefit effects on the cerebral vascular network in neonatal, adult and aged mice in control conditions. Indeed, little is known about hemodynamic responses of drugs used in preclinical studies in the developing brain as compared to adult rodents. To evaluate whether PJ34 may act on the NO pathway in the developing brain as in the peripheral circulatory system evidence these effects, we measured the hemodynamic effects including the cerebral vasoreactivity under CO_2_ in the absence and presence of PJ34 at the three developmental stages. We also measured the constitutive NOS activity, and the effect of non-selective and selective NOS inhibitors under PJ34.

## 2. Results

### 2.1. Cerebrovascular Reactivity (CVR) during Brain Development

In basal conditions, mean blood flow velocities (mBFVs) in the BT increased between P10 (13.3 ± 2.2 cm/s) and P12 (15.8 ± 1.6 cm/s, *p* = 0.0003; Table 1), in agreement with the expansion of the microvascular network [14]. The mBFVs measured at P12 were not different from those measured at P90 (14.6 ± 1.4 cm/s) and P365 (15.4 ± 1.9 cm/s), suggesting that the P12 age already represented a microvascular status close to that of adulthood. In controls and PBS-injected animals, there was an increase in mBFVs under CO_2_ reflecting the microvascular vasodilation, which represented a reserve of vasodilation of about 21%, 23%, 16%, and 26% in P10, P12, P90, and P365 mice, respectively (Table 1).

**Figure 1:**
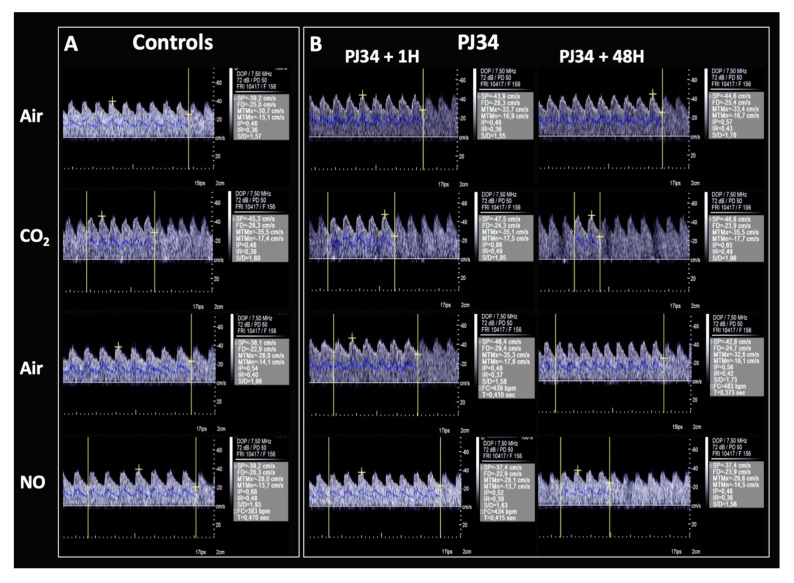
Representative Doppler velocity waveforms recorded in the basilar trunk (BT) in a control (**A**) and a PJ34-treated (**B**) adult mouse. Recordings were performed under light anaesthesia (isoflurane 0.5%) limiting the movement of the animal without any cardiorespiratory depression: (1) under air; (2) 3–5 min after breathing a gas mixture (CO_2_ 5%, O_2_ 16%, and N_2_ 79%) known to provoke a normoxia-hypercapnia in order to evaluate the cerebral arteriolar vasodilation mediated by the increase in metabolic demand and the NO-pathway named the cerebral vasodilation reserve; (3) under air; and (4) under air and 2–5 min after sublingual administration of a exogenous NO-donor in order to evaluate the cerebral vasodilation unmediated by the NO-pathway. (**A**) (Top) In the control mouse, the mean blood flow velocity (mBFV, blue line on the Doppler velocity waveform) increased under CO_2_ allowing the calculation of the cerebral vasodilation reserve in percentage (difference of mBFV under CO_2_ and air reported to mBFV under air). (Bottom) The mBFV under exogenous NO-donor decreased compared to only air condition representative of the BT dilation without any modification downstream of the arteriolar resistance (absence of modification of metabolic demand). (**B**) (right) One hour after PJ34 i.p. administration, the mBFV in the BT increased under air at a similar level than in the control animal under CO_2_, illustrative of the cerebral vasodilation properties of this pharmacologic agent. No additional increase in the mBFV was obtained under CO_2_, representative of the full recruitment on the cerebral vasodilation reserve. (Top left) the cerebral vasodilation persisted 48 h after PJ34 administration and (Bottom) the NO-donor was able to dilate the BT.

Under exogenous NO-donor, mBFVs increased about 32% and 21% in P10 and P12 mice, respectively, reflecting the maximal reserve of vasodilation. In the developing brain, the maximal mBFVs appeared similar to those measured under CO_2_ (P10: 16.1 ± 2.6 cm/s; P12: 19.4 ± 1.8 cm/s) or under exogenous NO (P10: 16.1 ± 3.3 cm/s; P12: 18.9± 3.2 cm/s). In contrast, in the P90 and P365 mice, mBFVs were lowered (from 14.5 ± 1.0 under air to 13.1 ± 0.8 cm/s under exogenous NO (*p* = 0.0028) and from 15.2 ± 1.2 under air to 13.4 ± 1.7 cm/s under exogenous NO (*p* = 0.0045), respectively) illustrative of the opposite responses of the cerebral micro- and macrovascular networks in adult brain compared to the developing brain (Table 1). Representative Doppler velocity waveforms recorded in the BT in a control and a PJ34-treated adult mouse are presented in Figure 1.

### 2.2. Effect of PJ34 on Cerebrovascular Reactivity

**Figure 2:**
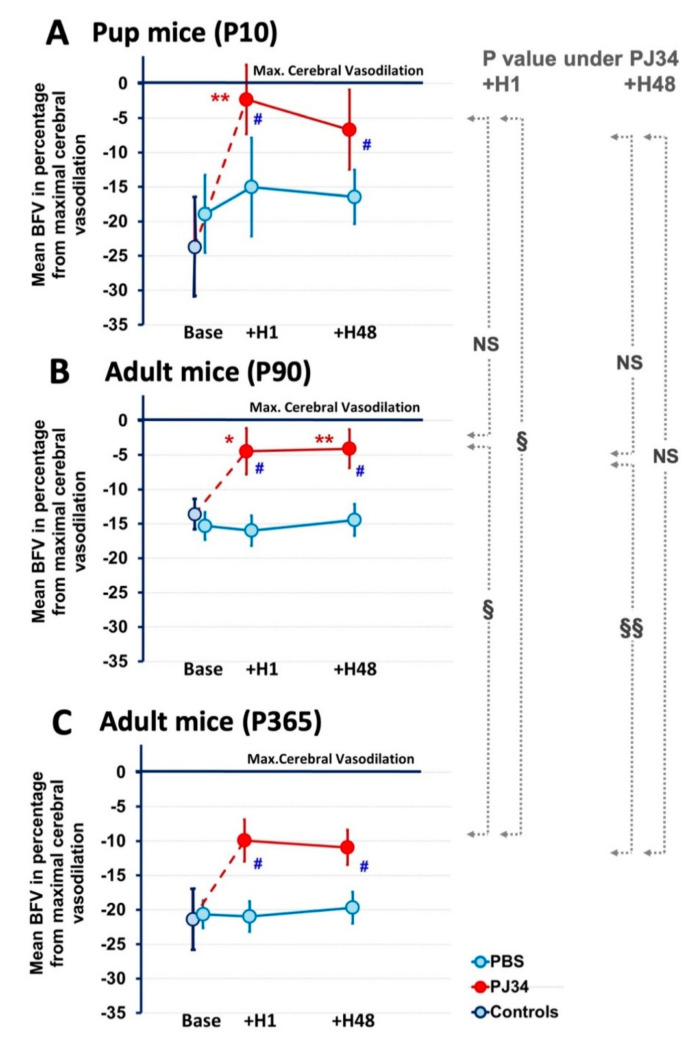
Vasoreactivity during development (P10 to P365) and effect of PJ34 on the maximal cerebral vasodilation in P10 (**A**), P90 (**B**) and P365 (**C**) mice. Data are represented as mean blood flow velocities (mBFVs) related to the maximal cerebral vasodilation under CO_2_ in basal (control-naive and PBS-treated) P10, P90, and P365 mice and 1 and 48 h after PJ34 administration. PJ34 induced a recruitment on the cerebral vasodilation reserve at each stage of development. At P365, the PJ34 vasodilator effect was lowered compared to P10 and P90. (* *p* < 0.005, ** *p* < 0.001 vs. basal, # *p* < 0.005, vs. PBS, § *p* < 0.005, §§ *p* < 0.001 PJ34 at +H1 or at +H48 between ages, *NS*: not significant).

PJ34 was i.p. injected to P10, P90, and P365 mice, and mBFVs were measured 1 and 48 h after to evaluate the vascular effect of PJ34 under air and/or 5% CO_2_ and compared to control mice (data are shown as percentage of the level of the basal mBFV reported to the maximal mBFV reached under CO_2_ in Figure 2, and in absolute values in Table 2), without any gender difference (see The Supplementary Materials, Table S1). We observed at 1 h that mBFVs measured under air for PJ34-treated P10 mice (15.0 ± 0.9 cm/s) dramatically increased and were similar to those measured under CO_2_ (15.3 ± 0.7 cm/s). Furthermore, CO_2_ was unable to more increase mBFVs measured under PJ34. This was illustrative of the whole recruitment of the cerebral vasodilator reserve mediated by PJ34 by itself. Again, 48 h after PJ34, mBFVs under air in P12 mice (19.1 ± 1.9 cm/s) were similar to those measured under CO_2_ (20.5 ± 1.4 cm/s, NS). These mBFVs values were elevated compared to those measured in controls under air (15.8 ± 1.6 cm/s, *p* = 0.0043), but similar to those measured in the controls under CO_2_ (19.4 ± 1.8 cm/s, NS) (Table 2 and Figure 2). In the P90 mice, we observed the same effects of PJ34 at both 1 and 48 h after injection, and no significant difference towards P10 and P90 mice was observed. In the P365 mice, this effect was also observed (Table 2 and Figure 2). However, under PJ34, the elevation of the mBFVs by recruitment on the cerebral vasodilator reserve was lesser extended compared to P90 mice. PJ34 did not modify HRs as compared to age-matched controls (Table 3).

### 2.3. PJ34 Cerebrovascular Reactivity is Mediated by NOS Activity

**Figure 3:**
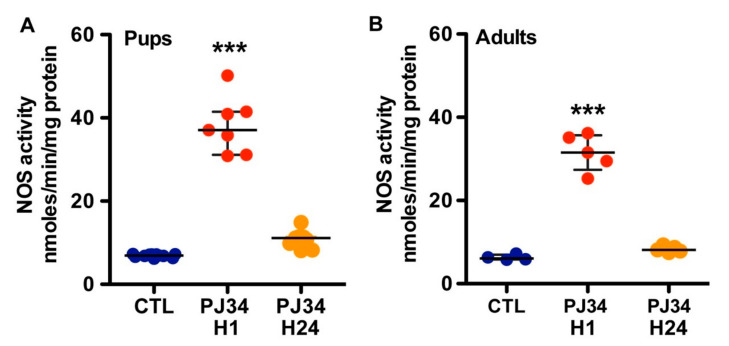
NOS activity in brain tissues from neonatal and adult mice in control conditions and after PJ34 administration (at 1 and 24 h). NOS activity in pup (**A**) and adult (**B**) mice is reported in nmol/min/mg protein and data were presented as the median and 25th–75th percentiles (*n* = 5–7). (*** *p* < 0.001 vs. the control (CTL) group).

To explain the vasodilation induced by PJ34, we then investigated the NO pathway because blood flow in the brain is regulated by neurons and astrocytes. As shown in Figure 3, PJ34 induced a significant increase in NOS activity in P10 mice (38.2 ± 6.7 nmol/min/mg protein; Figure 3A) as compared to age-matched control (6.9 ± 0.4 nmol/min/mg protein, *p* < 0.001) 1 h after administration. Twenty-four hours after PJ34 administration, NOS activity tended to remain enhanced (10.8 ± 2.0 nmol/min/mg protein compared to control, *p* < 0.05). In P90 mice, PJ34 also induced a significant increase in NOS activity (31.52 ± 4.40 nmol/min/mg protein; Figure 3B) as compared to age-matched control (6.29 ± 0.66 nmol/min/mg protein, *p* < 0.001) 1 h after administration. Then, NOS activity decreased (8.29 ± 0.74 nmol/min/mg protein) and did not differ from basal values.

### 2.4. Endothelial NOS Mediates the PJ34 Effects

**Figure 4:**
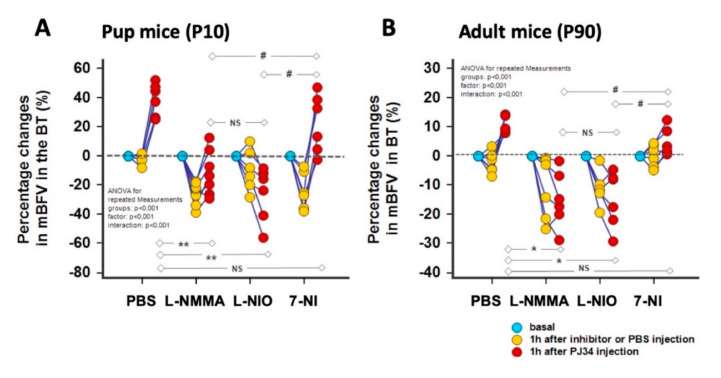
Relative changes in mean blood flow velocities (mBFVs) recorded in the basilar trunk (BT) versus basal states 1 h after PBS or specific or non-specific NOS inhibitors followed 1 h after by PJ34 injection. In P10 (**A**) and P90 (**B**) mice, L-NMMA was responsible of a decrease in mBFVs. L-NMMA as well as L-NIO abolished the increase in mBFVs consecutive to the PJ34 injection as shown in the PBS injected mice. In contrast, 7-NI did not prevent of this increase. Altogether, these finding lead to the mediation of the endothelial more than the neuronal NOS into the dynamic effects of PJ34. (* *p* < 0.005, ** *p* < 0.001 PBS + PJ34 vs. NOS inhibitors + PJ34; # *p* < 0.005 each NOS inhibitors + PJ34 between them, *NS*: not significant).

NOS inhibitors [15], at the doses used in this study (defined after pilot studies—see the Supplementary Materials), reduced mBFVs in the BT in P10 and P90 mice, except for 7-NI inhibitor in P90 mice. The PJ34 effect was abolished by L-NMMA in P10 (−11.2 ± 15.5%, *p* < 0.001) and P90 (−14.9 ± 9.7%, *p* < 0.001) mice, as compared to PBS-treated mice (+ 38.7 ± 11.2% in P10 and +10.4 ± 2.8% in P90 mice) (Figure 4). Similarly, L-NIO abolished the cerebral vasodilation after PJ34 injection (−26.1 ± 18.6%, *p* < 0.001; −14.8 ± 9.6%, *p* < 0.001, respectively, in pups and adult mice). In contrast, 7-NI followed by PJ34 injection permitted an increase in mBFVs of +22.2 ± 20% (*p* = 0.0048) in pups while mBFVs remained stable in adult mice +4.6 ± 4.8% (NS). Altogether these data suggest the role of the eNOS preferably to the nNOS into the cerebral vasodilation provided by PJ34 (Figure 4).

Although both sexes were included in our experiments, no differences about NOS activity, and effect of NOS inhibitors were observed between male and female mice.

## 3. Discussion

In this study, we observed that the PJ34 inhibitor is able by itself to enhance blood flow by recruitment on the microvascular vasodilation reserve, suggesting that PJ34 is likely to act on the endothelial function as well as during brain development than in adulthood.

Firstly, and in agreement with the development of the brain vasculature, we observed that blood flow velocities (BFVs) in the basilar trunk (BT) in P12 mice are similar to those measured in the adult, suggesting that P12 mice represents a developmental stage close to the adult. Active vascularisation persists at least through the third postnatal week in normal rat brain [14,16]. Vessel density increases in the normal rat cortex from P8 to P21 and is associated with endothelial cell proliferation during this period [17,18,19]. Expression of several vascular markers, such as the tight junction claudin-1, the basement membrane protein laminin, and the pericyte marker PDGFR-β increase in naive brains of P14 and P17 compared to P7 rat [14]. CO_2_-mediated vasoreactivity is impaired in the neonatal P7 rat, suggesting immaturity of the endothelial function [20] and appears in the juvenile P14 rat [21] similar to that measured in the adult.

**Figure 5:**
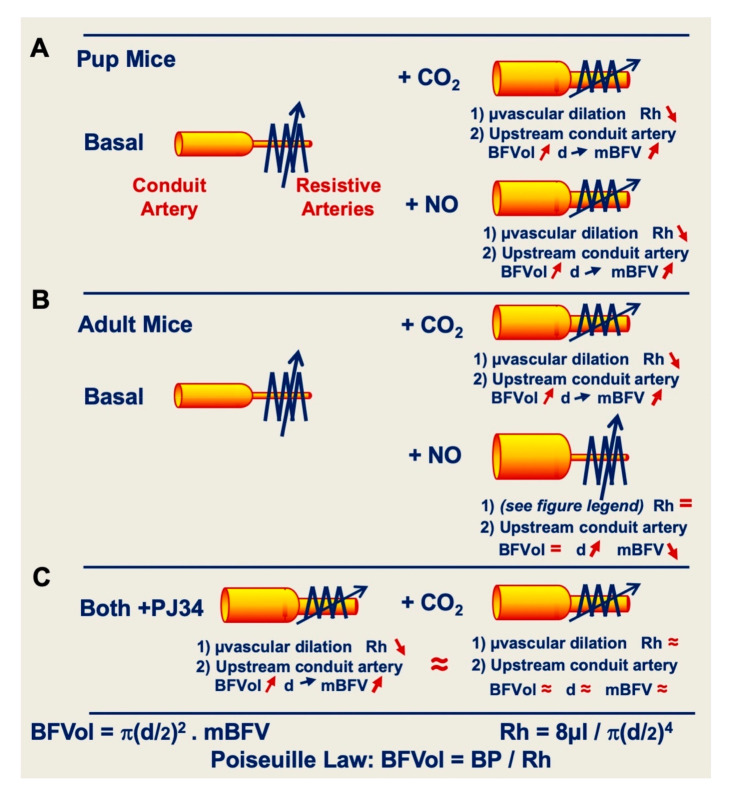
In an arterial network, the level of the hemodynamic resistance (Rh) inversely sets the value of the blood flow volume (BFVol) in the upstream conduit artery at each level of arterial blood pressure (BP) through the Poiseuille law. The level of the Rh is dependent of the inner diameter (d) of arterioles (resistive arteries). The BFVol determined, the mean blood flow velocity (mBFV) is adapted in relation to the section of the conduit artery. In pup (**A**) and adult **(B**) mice, CO_2_ gas mixture exposure provokes an arteriolar dilation with the consecutive upstream increase in the BFVol. Even if the conduit artery moderately dilates (5–8%, endothelial function), mBFVs increase illustrative of the downstream arteriolar dilation (20–40%, endothelial function) mediated by the metabolic command, representative of the cerebral vasoreactivity and the cerebral vasodilation reserve. In adults (**B**), an exogenous NO-donor provokes a dilation of the conduit artery without any modification of the level of Rh, thus of the BFVol, with a consecutive decrease in the mBFVs. In absence of metabolic modification, regulation of the microvascular tone is maintained by the sympathetic nervous system (SNS), which counteracts the NO-mediated arteriolar dilation. In the developing brain (**A**), SNS is immature and does not counteract the exogenous NO-mediated arteriolar dilation with the consecutive increase in BFVols and BFVs in the upstream conduit artery. In both adults and pup mice (**C**), 1 h after PJ34 administration, arterioles dilate and the BFVol and the mBFV increase in the upstream conduit arteries by recruitment on the cerebral vasodilation reserve without any significant additional effect under CO_2_.

During the CO_2_ gas mixture exposure in pup and adult mice, the increase in mBFV in the cerebral conduit arteries is due to the arteriolar dilation and consecutive cerebral blood flow (BF) increases mediated by the metabolic command through the induced hypercapnia. Even if the conduit arteries moderately dilate through the endothelial NO-pathway (endothelial function), the increase in cerebral BF was large enough to provoke a rise in mBFV in the upstream conduit arteries. This increase allowed us to evaluate the CVR quite equivalent at P12 and P90 stages (Figure 5A,B). In contrast, adult mice show a decrease in mBFV in cerebral conduit arteries under the exposure to an exogenous NO-donor, which is a well-established fact. NO-donors provoke a dilation of these arteries with a consecutive decrease in local mBFV because both CBF, and the downstream arteriolar resistance are maintained in absence of metabolic modification (Figure 5B). Previous studies showed the absence of metabolic stimulation illustrated by the absence of variation of the end-tidal partial pressure of CO_2_ and thus of the arterial blood gases before and during exogenous NO-donor, avoiding any confounding sources of dilation elicited by hypercapnia [22]. The putative mechanism for the maintenance of arteriolar resistance could be the opposite action of the sympathetic nervous system (SNS) with proportionately greater vasoconstrictor effects on the downstream arterioles than on the conduit arteries. The arteriolar constriction mediated by the SNS thereby enters in competition with the dilatory effect of the delivered exogenous NO leading to the absence of hemodynamic arteriolar resistance and cerebral BF in absence of metabolic stimulation. It was reported previously that an exogenous NO-donor leads to a reflexive increase in plasma noradrenaline to counteract the drug-induced hypotension [23]. Moreover, intravenous NO-donor administration in rat and guinea pig has been reported to mimic biological responses associated with sympathetic neuronal activity in perfused atria and cerebrospinal fluids. Numerous studies [23,24,25,26] indicate that neurogenic arteriolar vasoconstriction might be one potential compensatory mechanism to counteract the drug-induced arteriolar dilation in order to maintain the cerebral BF in absence of metabolic modification (see above). However, in foetuses and neonates, the absence of a significant alteration in arterial pressure during sympathectomy suggests a minor role for the adrenergic system in the maintenance of resting tone of peripheral circulation [27]. In our pup mice, the SNS is thus immature and cannot counteract the arteriolar dilation mediated by an exogenous NO-donor. The CBF thus increased with the consecutive increase in mBFV in the upstream cerebral conduit arteries even if it moderately dilates (endothelial function) (Figure 5A). Nevertheless, pup and adult mice showed an actual cerebral arteriolar dilation 1 h after PJ34 administration by recruitment on the cerebral vasodilation reserve without any additional effect under CO_2_ gas mixture inhalation (Figure 5C).

PJ34 by itself was able to increase cerebral BF by recruitment on the cerebral vasodilation reserve. This increase is nitric oxide (NO) dependent under the activation of the Ca^2+^-dependent constitutive NO synthases (cNOS). NO is a powerful vasodilator involved in both physiological functions and pathophysiological alterations, namely the maintenance of vascular tone. In the brain, several lines of evidence support a key role of neuronal NOS (nNOS) in BF regulation, which produces much of the NO as compared to smaller endothelial NOS (eNOS)-derived NO amounts [28,29].

1. Using NOS inhibitors in the P10 mice, namely L-NMMA (a pan inhibitor) and 7-NI (a more selective nNOS inhibitor), the reduction in BFV appears similar, suggesting that nNOS is important in the neonatal brain for vasodilation compared to the eNOS, and that L-NMMA exerts preferentially its effect through the inhibition of nNOS. Using L-NIO, the reduction in BFV is not so drastic at 1 h after injection, but this inhibitor completely reverses the PJ34 effect.
2. In contrast, in the adult mice, L-NMMA mainly blocks the eNOS (at a similar level obtained with L-NIO), and both inhibitors counteract the PJ34 effect.
3. Finally, 7-NI reduces BFVs in the P10 brain, in agreement with key role of nNOS in BF regulation [30], which is not the case in the adult brain. However, this inhibitor does not counteract the PJ34 effect in both neonatal and adult brain.

PARP has been implicated in endothelial dysfunction in various diseases such as atherosclerosis, hypertension, diabetes, chronic heart failure, and aging [31]. In adult Wistar rats, PARP enzyme is activated in the endothelium after endothelin-1 (ET-1) incubation, and PJ34 counteracts ET-1-induced endothelial dysfunction [11]. Furthermore, PARP activation contributes to impaired eNOS-mediated vasodilation of aortas from diabetic animals [32], and to nNOS dysfunction in the maximum relaxation of in vitro gastric fundus in diabetic rats [33]. PARP inhibition (with the 3-aminobenzamide inhibitor) partially corrected the maintenance of relaxation responses [33] and was able to restore erectile function by activation of NO/cGMP pathway in diabetic rats [34]. Treatment with PJ34 significantly prevented the decrease in cNOS activity measured in corpus cavernosa in diabetic adult SD rats [35]. PJ34 was shown to act synergistically with recombinant tissular—plasminogen activator to improve reperfusion and reduce middle cerebral artery vasospasm in a thromboembolic model of cerebral ischemia [36]. Altogether, these data highly suggest that PJ34 may represent a powerful compound to restore NOS dysfunction in cardio- and cerebrovascular diseases. Our study suffers from some limitations, because in vitro study (vasoconstriction and vasodilation) on isolated cerebral arteries, middle cerebral artery, or basilar trunk) cannot be investigated. These arteries are too small and thin as compared to the currently used peripheral artery, for instance mesenteric artery, to be withdrawn without any partial lesion, even more in mouse pup brain. Moreover, in vitro study cannot mimic the neurovascular coupling.

## 4. Material and Methods

### 4.1. Ethics Statement

All experiments were performed in accordance with the European Community guidelines (Directive 2010/63/EU) and the French National guidelines for the care and use of laboratory animals. All animal procedures were approved by the local Ethics Committee in Animal Experimentation (protocol number APAFiS # 03560.02; 19 January 2018), and in compliance with the ARRIVE guidelines (https://www.nc3rs.org.uk/arrive-guidelines).

### 4.2. Animals and Inclusion/Exclusion

C57Bl6/J neonatal (P10, *n* = 59), adult (P90, *n* = 55) and aged (P365, *n* = 12) mice (both sexes) were purchased from Janvier Labs (Le Genest St-Isle, France). All animals were included in this study (no mortality) and randomly allocated to the different sets of experiments.

### 4.3. Drug and Study Design

**Figure 6:**
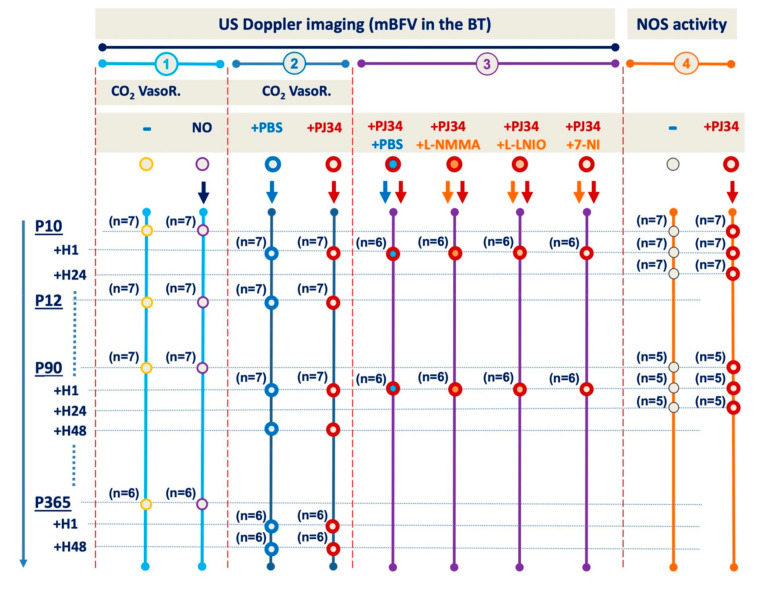
Outline of the experimental procedure in C57Bl/6j mice subjected to cerebral vasoreactivity in absence and/or presence of PJ34 and in presence of NOS inhibitors.

The study design is reported in Figure 6.

In the first set of experiments, neonatal (at 2 developmental stages P10 and P12, *n* = 7 each), adult (P90, *n* = 7), and aged (P365, *n* = 6) mice were subjected to US Doppler measures (see below) under air, CO_2_ gas mixture inhalation, and then return to under air. Fifteen minutes later, neonatal and adult mice received a single administration of an exogenous NO-donor sublingually (50 µL, Natispray^®^ 0.15 mg/67.5 µL) and were subjected to US Doppler measures five minutes later. All these measures were done in basal conditions.

In the second set of experiments, P10 (*n* = 14), P90 (*n* = 14) and P365 (*n* = 12) mice were i.p. administered with either phosphate buffered saline (PBS) or PJ34 (10 mg/kg, Sigma-Aldrich, France [8], and then US Doppler measures were performed at 1 and 48 h after. Other mice (neonatal and adult, control and PJ34-injected) were used to obtain brain tissues for both NOS activity and RT-qPCR (at 1 and 24 h after administration, *n* = 5–7).

In the last set of experiments, P10 and P90 mice received commonly recognised NO synthase (NOS) inhibitors: L-NMMA (Alexis, Enzo Life Sciences, USA, #106-001M025; inhibiting both neuronal NOS (nNOS) and eNOS at a similar IC_50_ [15]), L-NIO (Calbiochem, France, CAS #159190-44-0; inhibiting more selectively eNOS) [30]; (or 7-NI (Sigma Aldrich, France, #N7778; inhibiting more selectively nNOS [15]. Mice received L-NMMA at 30 or 20 mg/kg, respectively, at P10 and P90. L-NIO and 7-NI were administered respectively at 10 and 5 mg/kg, in both P10 and P90 mice (for the doses, see Figure S1). Others age-matched mice (at both ages) received PBS. One hour after NOS inhibitors or PBS administration, mice received a single PJ34 injection (i.e., at 10 mg/kg). Blood velocities were measured in basal conditions, 1 h after NOS inhibition, and 1 h after PJ34 administration.

### 4.4. Ultrasound Imaging

Thermoregulated mice were subjected to ultrasound measurements under isoflurane anaesthesia (0.3% for pups to 0.5% for adults) using an echocardiograph (ACUSSON S3000, Siemens, Erlangen, Germany) equipped with a 14.5-MHz linear transducer [37]. Time-average mean blood flow velocities (mBFVs) were measured in the basilar trunk (BT) in different conditions (see below). Heart rates (HRs) were measured and reflected changes in cardiac output, as ventricular volume is quite invariable in newborns. The mBFV measurements were investigated in the 7 pup and 7 adult control mice at basal states for intra- and inter-observer repeatability. Two series of measurements separated by a 10 min interval were recorded. The repeatability coefficient (RC) was calculated as defined by the British Standard Institution [38], i.e., according to the formula RC^2^ = ∑ Di^2^/*n*, where Di is the relative (positive or negative) difference within each pair of measurements and *n* is the sample. The intra-observer and inter-RC values were 0.5 and 0.7 cm/s for pup mice, 0.4 and 0.5 cm/s for adult mice largely inferior to the differences exhibited between the different groups when considered as statistically significant.

### 4.5. Cerebrovascular Reactivity (CVR) to Carbon Dioxide (CO_2_) and/or to Exogenous NO-Donor

Heart rate, peak systolic, end-diastolic, and time-averaged mean blood flow velocity (mBFV) were measured in the BT, first in basal normoxic-normocapnic conditions, and then in normoxic-hypercapnic conditions, i.e., during inhalation through the mask of a gas mixture containing 5% CO_2_, 16% O_2_, and 79% N_2_ flowing through the vaporiser to maintain the slight anaesthesia for 3–5 min [39]. Vasoreactivity was then estimated as a percentage increase in mBFV recorded in hypercapnia compared to the basal mBFV recorded in normocapnia. Five to ten minutes after returning to ambient air, Doppler recordings in the BT were repeated to verify the return of the mBFV to basal values. Fifty microlitres of NO-donor (Natispray^®^) was sublingually delivered to the animal. BFVs were then recorded every minute over 5 min, and the maximal mBFV reached was chosen and reported to the basal value to estimate the percentage of cerebral vasoreactivity under exogenous NO-donor. All these measures were done in animals without any PBS injection.

### 4.6. Arterial Blood Pressure and Blood Gases Analysis

Systolic arterial blood pressure (BP) was monitored, in 7 anaesthetised adult mice (isoflurane 0.5%) under air and/or CO_2_ gas mixture, using a tail-cuff plethysmograph connected to a computerised system (BP-2000 Blood Pressure Analysis System; Visitech Systems, Apex, NC, USA). Arterial blood was then sampled by ultrasonography-guided puncture of the left heart ventricle. Blood was collected in heparinised tubes and immediately examined for blood gases analysis using an ABL90 Series blood gas analyser (Radiometer Medical ApS, Brønshøj, Denmark). Table 4 reports BPs and blood gases data showing the actual hypercapnic and normoxic respiratory conditions obtained under 5% CO_2_, 16% O_2_, and 79% N_2_, without any modification of the BPs.

### 4.7. NOS Activity

NOS activity was measured by monitoring the conversion of tritium-labelled L-arginine to tritium-labelled L-citrulline as previously described [40], and according to Manivet et al. [41].

### 4.8. Statistical Analysis

For Doppler ultrasound measurements, continuous variables (HRs, BPs, blood gases, and mBFVs) are reported as mean ± standard deviation (SD). The Gaussian distribution was assessed using the D’Agostino–Pearson test in all groups. Continuous variables were analysed with a two-way ANOVA according to mouse’s age and treatment, and at each age with an ANOVA for repeated measurement according to the time-points of the protocol and the drugs injected. Delta variations and percentage variations in mBFV for each mouse between the different time-points of the different applied protocols were calculated compared to basal values. Statistics were then performed on delta-variations values, not on the percentage values. When ANOVAs were significant, differences between treatments’ groups were then evaluated with post hoc Bonferroni and unpaired or paired Student t-test as required (MedCalc^®^ Statistical Software version 18.2.1, MedCalc Software bvba, Ostend, Belgium). In all cases, the power of the tests was verified and considered as significant when > 80% (http://www.anastats.fr, ANASTATS, Rilly Sur Vienne, France). Only *p* values < 0.005 were considered as significant [42]. As Gaussian distribution was absent for NOS activities, data were expressed as median ± interquartile range. Values were compared using one-way ANOVA, and a post hoc Newman–Keuls test to analyse differences between two groups using PRISM 5 software (GraphPad, San Diego, CA, USA). *p* values < 0.01 were considered as significant.

## 5. Conclusions

The current study provides non-invasively, in the entire animal, the first evidence that PARP inhibition with PJ34 inhibitor is able to increase cerebral BF through the stimulation of the NO-pathway. The amplitude of the NO-mediated blood supply recruitment through the collateral circulation to the ischemic penumbra is a powerful predictor of stroke outcome in both infant and adult stroke [13]. In that way, PJ34 can represent an interesting agent for the treatment of ischemic cerebrovascular diseases, during which the establishment of collateral recruitment in the penumbra is more or less impaired because of a current endothelial dysfunction, namely in patients with diabetes mellitus or hypertension, at higher risk for stroke incidence.

## Figures and Tables

**Table 1:** Mean blood flow velocities (mBFVs) recorded in the basilar trunk in control (naive) mice at different developmental stages under air, 5% CO_2_ - 16% O_2_ - 79% N_2_, and/or after exogenous NO-donor sublingual administration.

	Mean BFV (cm/s)	Air	CO_2_	*p* Value (CO_2_ vs. Air)	Air	NO	*p* Value (NO vs. Air)
Pup Mice	P10 (*n* = 7)	13.3 ± 2.2	16.1 ± 2.6	0.0016	12.2 ± 2.3	16.1 ± 3.3	0.0007
	P12 (*n* = 7)	15.8 ± 1.6	19.4 ± 1.8	0.0011	15.6 ± 2.0	18.9 ± 3.2	0.0034
	*p* value P12 vs. P10	0.0003	0.0001		0.0003	0.0035	
Adult Mice	P90 (*n* = 7)	14.6 ± 1.4	16.9 ± 1.5	<0.0001	14.5 ± 1.0	13.1 ± 0.8	0.0028
	*p* value P90 vs. P12	NS	NS		0.0002	NA	
	P365 (*n* = 6)	15.4 ± 1.9	19.4± 2.0	<0.0001	15.2 ± 1.2	13.4 ± 1.7	0.0045
	*p* value P365 vs. P90	NS	NS		NS	NS	

Significant threshold was set at *p* < 0.005. NS: not significant. NA: not applicable.

**Table 2:** Mean blood flow velocities recorded in the basilar trunk in control (naive) and PJ34-treated mice at different developmental stages under air and/or 5% CO_2_ - 16% O_2_ - 79% N_2_.

Mean BFV (cm/s)			Basal	PJ34 + H1	*p* Value (PJ34 vs. Basal)	PJ34 + H48	*p* Value (PJ34 vs. Basal)
Pup Mice	P 10 (*n* = 7)	Air	13.3 ± 2.2	15.0 ± 0.9	NS		
		CO_2_	16.1 ± 2.6	15.3 ± 0.7	NS		
		*p* value CO_2_ vs. air	0.0016	NS			
	P 12 (*n* = 7)	Air	15.8 ± 1.6			19.1 ± 1.9	0.0043
		CO_2_	19.4 ± 1.8			20.5 ± 1.4	NS
		*p* value CO_2_ vs. air	0.0011			NS	
Adult Mice	P 90 (*n* = 7)	Air	14.6 ± 1.4	15.0 ± 0.8	NS	16.1 ± 0.9	0.0049
		CO_2_	16.9 ± 1.5	15.8 ± 0.7	NS	16.7 ± 0.5	NS
		*p* value CO_2_ vs. air	<0.0001	NS		NS	
	P 365 (*n* = 6)	Air	15.7± 1.7	17.7 ± 1.2	0.0033	17.5 ± 2.0	0.0024
		CO_2_	19.7 ± 1.7	19.6 ± 0.7	NS	19.6 ± 2.2	NS
		*p* value CO_2_ vs. air	<0.0001	<0.0001		0.0002	

Significant threshold was set at *p* < 0.005. NS: not significant.

**Table 3:** Heart rate in control (naive) and PJ34-treated mice at different developmental stages under air and/or 5% CO_2_ - 16% O_2_ - 79% N_2_.

Heart Rate (bpm)			Basal	PJ34 + H1	*p* Value (PJ34 vs. Basal)	PJ34 + H48	*p* Value (PJ34 vs. Basal)
Pup Mice	P 10 (*n* = 7)	Air	431 ± 62	526 ± 61	NS		
		CO_2_	340 ± 44	413 ± 37	NS		
		*p* value CO_2_ vs. air	0.0003	0.0010			
	P 12 (*n* = 7)	Air	546 ± 61			603 ± 27	NS
		CO_2_	480 ± 39			497 ± 32	NS
		*p* value CO_2_ vs. air	NS			0.0001	
Adult Mice	P 90 (*n* = 7)	Air	465 ± 64	438 ± 43	NS	474 ± 14	NS
		CO_2_	443 ± 45	450 ± 17	NS	489 ± 52	NS
		*p* value CO_2_ vs. air	NS	NS		NS	
	P 365 (*n* = 6)	Air	481 ± 65	486 ± 39	NS	489 ± 12	NS
		CO_2_	496 ± 73	515 ± 31	NS	496 ± 73	
		*p* value CO_2_ vs. air	NS	NS		NS	

Significant threshold was set at *p* < 0.005. NS: not significant. Note the classical bradycardia during hypercapnia in immature animals.

**Table 4:** Systolic arterial blood pressures (BP), heart rates (HR) and pH, arterial CO_2_ and O_2_ partial pressures and O_2_ saturation in anaesthetized (0.5% isoflurane) adult control mice under air and/or 5% CO_2_-16% O_2_-79% N_2_.

	Systolic BP (mmHg)	HR (bpm)	pH	pCO_2_ (mmHg)	pO_2_ (mmHg)	SatO_2_ (%)
Air	82 ± 11	465 ± 64	7.35 ± 0.10	29.5 ± 6.1	90.7 ± 18.8	91.9 ± 10.5
CO_2_	81 ± 10	443± 45	7.17 ± 0.09	46.8 ± 9.3	84.2 ± 11.9	81.1 ± 13.4
*p* value (CO_2_ vs. Air)	NS	NS	0.0036	0.0014	NS	NS

Significant threshold was set at *p* < 0.005. NS: not significant.

## Supplementary Materials

Supplementary materials can be found at https://www.mdpi.com/1422-0067/21/18/6569/s1.
