# Supplementary material for: Cerebral Vasodilator Property of Poly(ADP-Ribose) Polymerase Inhibitor (PJ34) in the Neonatal and Adult Mouse Is Mediated by the Nitric Oxide Pathway
Source: Int J Mol Sci. 2020 Sep 8;21(18):6569. doi: 10.3390/ijms21186569 (PMC7555622; doi:10.3390/ijms21186569)
Supplement: Supplementary file 1 [file ijms-21-06569-s001.pdf]

Online supplement

Cerebral Vasodilator Property of Poly(ADP-Ribose) Polymerase Inhibitor (PJ34) in the Neonatal and Adult Mouse is Mediated by the Nitric Oxide Pathway Philippe Bonnin, Christiane Charriaut-Marlangue, Julien Pansiot, Alexandre Boutigny, Jean-Marie Launay and Valérie C Besson.

Materials and Methods

NO synthase inhibitors. In a pilot study in P10 mice we observed that 20 mg/kg L-NMMA was not able to induce, at least, a 50% reduction in blood flow (Figure) as previously demonstrated in the P7 rat pup [30]. In contrast, this dose was efficient in P90 mice. Using a 30 mg/kg dose, cerebral blood flow was significantly reduced (see Figure 4A in the manuscript).

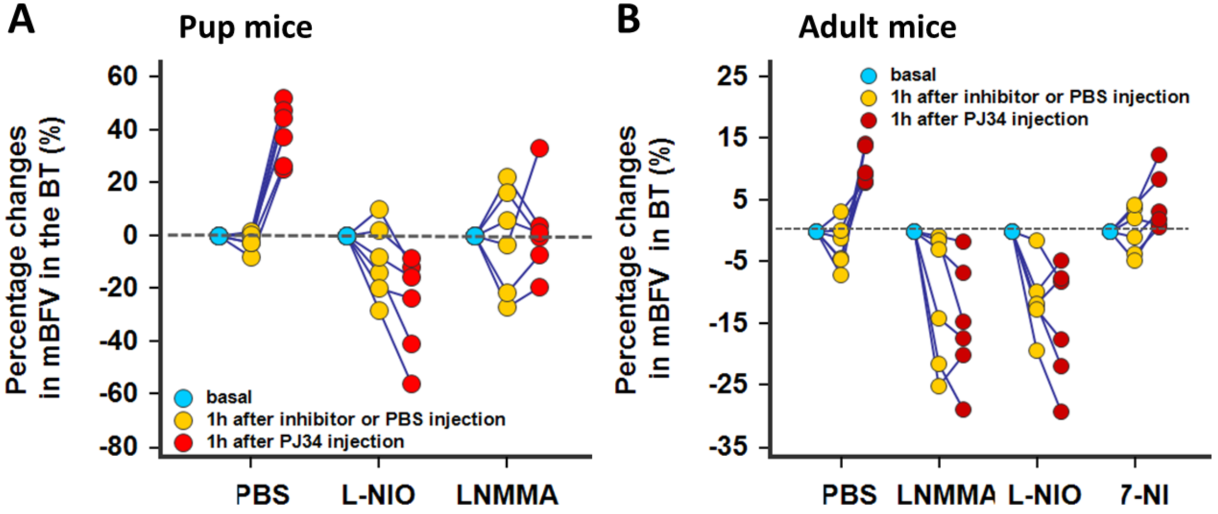

In another pilot study, whereas 7-NI at 5 mg/kg was able to reduce cerebral blood flow in P10 mice, this dose did not reduce blood flow in the adult mice whatever the dose used. Furthermore, it is well known that 7-NI at high doses inhibits both neuronal and endothelial NO synthase.

NOS activity. Briefly, after sonication, thawed tissues were centrifuged at 4°C firstly at 2,000 x g for 10 min and then at 10,000 x g for 15 min. The supernatants (30 µL) were incubated in the presence and absence of nitro-L-arginine (1 mM) for 30 min at 37°C with L-[^{3}H]arginine (1 µM; 1 µCi), 1 mM NADPH, 15 µM (6R)-5,6,7,8-tetrahydro-L-biopterin, 1 µM flavine-adenine dinucleotide, and 1 µM calmodulin in 50 mM HEPES buffer (pH 7.40) containing 1mM DL-dithiothreitol, 1 mM EDTA, and 1.25 mM $CaCl_2$ (final volume, 150 µL). Incubations were quenched by the addition, for 5 min at 4°C, of 1 ml of ice-cold 100 mM HEPES buffer (pH 5.50) containing 10 mM EGTA and 500 mg of AG 50W-X8 (counter-ion Na^+) cation exchange resin, followed by a 5-min centrifugation at 10,000 x g. L-[^{3}H]Citrulline in the supernatant was quantified by liquid scintillation counting. Specific NOS activity was calculated as the nitro-L-arginine-sensitive formation of L-[^{3}H]citrulline/min/mg of protein. L-Citrulline was quantitatively recovered by this batch assay as determined by using L-[^{14}C]citrulline. L-[2,3,4-^{3}H]arginine monohydrochloride (1.84 TBq/mmol), and L-[^{14}C]citrulline (1.73 GBq/mmol) were from Perkin-Elmer NEN Life Science Products. Other chemicals, of the purest grade available, were from Sigma-Aldrich.

Table S1: Mean blood flow velocities recorded in the basilar trunk in control (naive) and PJ34-treated mice at different developmental stages under air and/or 5% CO_2 - 16% O_2 - 79% N_2, with sex differentiation.

		Mean BFV (cm/s)	Basal				PJ34 + H1				P value (PJ34 *vs* Basal)	PJ34 + H48				P value (PJ34 *vs* Basal)
			**All**	***Females***		***Males***	**All**	***Females***		***Males***		**All**	***Females***		***Males***	
**Pup Mice**	**P 10 (n=7)**		**(n=7)**	***(n=3)***		***(n=4)***	**(n=7)**	***(n=5)***		***(n=2)***						
		**Air**	13.3 ± 2.2	*13.1 ± 2.0*	*NS*	*13.4 ± 2.6*	15.0 ± 0.9	*14.8 ± 1.0*	*NS*	*15.5 ± 0.9*	*NS*					
		**CO_2**	16.1 ± 2.6	*15.9 ± 0.4*	*NS*	*16.2 ± 3.7*	15.3 ± 0.7	*15.3 ± 0.7*	*NS*	*15.9 ± 0.9*	*NS*					
		P value CO_2 vs air	*0.0016*				*NS*									
	**P 12 (n=7)**		**(n=7)**	***(n=3)***		***(n=4)***						**(n=7)**	***(n=5)***		***(n=2)***	
		**Air**	15.8 ± 1.6	*15.8 ± 2.3*	*NS*	*15.8 ± 0.9*						19.1 ± 1.9	*18.8 ± 2.3*	*NS*	*19.9 ± 0.2*	*0.0043*
		**CO_2**	19.4 ± 1.8	*18.9 ± 2.5*	*NS*	*19.9 ± 0.9*						20.5 ± 1.4	*20.5 ± 1.7*	*NS*	*20.4 ± 0.1*	*NS*
		P value CO_2 vs air	*0.0011*									*NS*				
**Adult Mice**	**P 90 (n=7)**		**(n=7)**	***(n=3)***		***(n=4)***	**(n=7)**	***(n=3)***		***(n=4)***		**(n=7)**	***(n=3)***		***(n=4)***	
		**Air**	14.6± 1.4	*13.5 ± 0.9*	*NS*	*15.4 ± 1.3*	15.0 ± 0.8	*14.6 ± 0.8*	*NS*	*15.4 ± 0.8*	*NS*	16.1 ± 0.9	*15.6 ± 0.6*	*NS*	*16.4 ± 0.8*	*0.0049*
		**CO_2**	16.9 ± 1.5	*15.7 ± 0.7*	*NS*	*17.8 ± 1.4*	15.8 ± 0.7	*15.5 ± 2.2*	*NS*	*15.9 ± 0.6*	*NS*	16.7 ± 0.5	*16.5 ± 0.4*	*NS*	*17.0 ± 0.5*	*NS*
		P value CO_2 vs air	*<0.0001*				*NS*					*NS*				
	**P 365 (n=6)**		**(n=6)**	***(n=3)***		***(n=3)***	**(n=6)**	***(n=3)***		***(n=3)***		**(n=6)**	***(n=3)***		***(n=3)***	
		**Air**	15.7± 1.7	*15.9 ± 2.6*	*NS*	*15.5 ± 0.8*	17.7 ± 1.2	*18.3 ± 1.3*	*NS*	*17.0 ± 0.8*	*0.0033*	17.5 ± 2.0	*18.4 ± 2.2*	*NS*	*16.5 ± 1.7*	*0.0024*
		**CO_2**	19.7 ± 1.7	*19.6 ± 2.4*	*NS*	*19.8 ± 1.1*	19.6 ± 0.7	*19.9 ± 0.8*	*NS*	*19.4 ± 0.7*	*NS*	19.6 ± 2.2	*20.7 ± 2.6*	*NS*	*18.6 ± 1.5*	*NS*
		P value CO_2 vs air	*<0.0001*				*<0.0001*					*0.0002*				

Significant threshold was set at $p<0.005$. *NS*: not significant.
